# Non-isothermal kinetics of thermal degradation of chitosan

**DOI:** 10.1186/1752-153X-6-81

**Published:** 2012-08-02

**Authors:** Velyana Georgieva, Dilyana Zvezdova, Lyubomir Vlaev

**Affiliations:** 1 Department of Physical Chemistry, Assen Zlatarov University, Burgas, 8010, Bulgaria; 2 Department of Organic Chemistry, Assen Zlatarov University, Burgas, 8010, Bulgaria

**Keywords:** Chitosan, Thermal degradation, Non-isothermal kinetics, Kinetics triplet

## Abstract

****Background**:**

Chitosan is the second most abundant nitrogen containing biopolymer in nature, obtained from the shells of crustaceans, particularly crabs, shrimp and lobsters, which are waste products of seafood processing industries. It has great potential application in the areas of biotechnology, biomedicine, food industries, and cosmetics. Chitosan is also capable of adsorbing a number of metal ions as its amino groups can serve as chelation sites. Grafted functional groups such as hydroxyl, carboxyl, sulfate, phosphate, and amino groups on the chitosan have been reported to be responsible for metal binding and sorption of dyes and pigments. The knowledge of their thermal stability and pyrolysis may help to better understand and plan their industrial processing.

****Results**:**

Thermogravimetric studies of chitosan in air atmosphere were carried out at six rates of linear increasing of the temperature. The kinetics and mechanism of the thermal decomposition reaction were evaluated from the TG data using recommended from ICTAC kinetics committee iso-conversional calculation procedure of Kissinger-Akahira-Sunose, as well as 27 mechanism functions. The comparison of the obtained results showed that they strongly depend on the selection of proper mechanism function for the process. Therefore, it is very important to determine the most probable mechanism function. In this respect the iso-conversional calculation procedure turned out to be the most appropriate.

****Conclusion**:**

Chitosan have excellent properties such as hydrophilicity, biocompatibility, biodegradability, antibacterial, non-toxicity, adsorption application. The thermal degradation of chitosan occurs in two stages. The most probable mechanism function for both stages is determined and it was best described by kinetic equations of *n*^-th^ order (*F*_n_ mechanism). For the first stage, it was established that *n* is equal to 3.0 and for the second stage – to 1.0 respectively. The values of the apparent activation energy *E*, pre-exponential factor *A* in Arrhenius equation, as well as the changes of entropy Δ*S*^≠^, enthalpy Δ*H*^≠^ and free Gibbs energy Δ*G*^≠^ for the formation of the activated complex from the reagent are calculated.

## Background

A number of reviews [1-9] and articles [10-15] have been dedicated to chitin and the products obtained from its chemical treatment at different conditions. Chitosan is the second most abundant nitrogen containing biopolymer in nature, obtained from the shells of crustaceans, particularly crabs, shrimp and lobsters, which are waste products of seafood processing industries [13,15].

**Figure 1:**
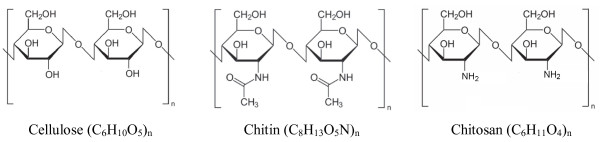
Structures of cellulose, chitin and chitosan.

Chitosan, namely poly-β(1–4)-2-amino-2-deoxy-*D*-glucopyranose is a natural high molecular mass biopolymer which is generally obtained by extensive deacetilation of chitin [poly-β(1–4)-2-acetamide-2-deoxy-*D*-glucopyranose] [10]. It occurs as a component of the cell wall of some fungi but it is generally produced by carrying out the deacetilation of chitin. A sharp nomenclature with respect to the degree of N-deacetilation has not been defined between chitin and chitosan. The major procedure for obtaining chitosan is based on the alkaline deacetylation of chitin with strong alkaline solution. When deacetilation is incomplete a mixture of acetamide-polymer and amino-polymer are obtained producing materials with different properties called chitosans. Degree of deacetilation of 70-80% is very common. The degree of deacetylation and the crystallinity of chitosans are the principal characteristics affecting both solubility in aqueous medium and capacity for forming complexes [14]. The structure of cellulose, chitin and chitosan are shown in Figure 1.

Chitin and chitosan are of commercial interest due to their high percentage of nitrogen (6.89%) compare to synthetically substituted cellulose (1.25%). In this respect, chitin and chitosan are recommended as suitable functional materials, because these natural polymers have excellent properties such as hydrophilicity, biocompatibility, biodegradability, antibacterial, non-toxicity, adsorption properties, and remarkable affinity for many biomacromolecules. Chitosan has been widely studied for biosensors, tissue engineering, separation membrane, waste water treatment, because of its multiple functional groups [15].

Recently, much attention has been paid to chitin and chitosan as a potential polysaccharide resource. Due to their special chemical and biological properties and widespread availability chitins and their derivatives have extensive applications in many industrial, and agricultural fields [15,16]. Chitosan have great potential application in the areas of biotechnology, biomedicine, food industries, and cosmetics. Chitosan is also capable of adsorbing a number of metal ions as its amino groups can serve as chelation sites. Due to their high nitrogen content and porosity, chitosan-based sorbents have exhibited relatively high sorption capacities and kinetics for most heavy metal ions. This could be easily explained by the presence of amino groups in the polymer matrix, which can interact with metal ions in the solution by ion exchange and complexation reactions [8,15]. The high content of amino groups also makes possible many chemical modifications in the polymer with the purpose of improving its sorptive features, such as selectivity and adsorption capacity. Grafted functional groups such as hydroxyl, carboxyl, sulfate, phosphate, and amino groups on the chitosan have been reported to be responsible for metal binding and sorption of dyes and pigments [17-22]. The knowledge of their thermal stability and pyrolysis may help to better understand and plan their industrial processing.

Studies on mechanism and kinetics of reactions involving solid compounds is challenging and difficult task with complexity results from the great variety of factors with diverse effects, e.g. reconstruction of solid state crystal lattice, formation and growth of new crystallization nuclei, diffusion of gaseous reagents or reaction products materials heat conductance, static or dynamic character of the environment, physical state of the reagents – dispersity, layer thickness, specific area and porosity, type, amount and distribution of the active centers on solid state surface, etc. [23]. Recently, the methods of thermogravimetry (TG), differential thermal analysis (DTA) and differential scanning calorimetry (DSC) are quite useful, since they provide reliable information on the physico-chemical parameters, characterizing the processes of transformation of solids or participation of solids in processes of isothermal or non-isothermal heating [23-28]. The kinetic triplet (i.e., *EA* and *f*(*α*)) is typical outcome of the regular kinetic analysis. Practically, the kinetic triplet is needed to provide mathematical description of the process. If the kinetic triplet is determined correctly, it can be used reproduce the original kinetic data as well as to predict the process kinetics outside the experimental temperature region. For example, may be predicting at any desired temperature, the time to reach any extent of conversion [29]. The results, obtained on this basis can be directly applied in materials science for the preparation of various metals and alloys, cements, ceramics glasses, enamels, glazes, synthetic or natural polymers and composite materials [23]. In the case of chitosan, several studies concerning the thermal degradation by means of TG, DTA and DSC techniques were reported and different kinetic values were obtained depending on the experimental conditions in which essay were performed [12-14,30,31]. The knowledge of thermal degradation kinetic may help a better understanding and planning of processes to recover metals or metal oxides previously adsorption on chitosan.

The aim of this paper is to assess the kinetic models of chitosan pyrolysis and to estimate the kinetics triplet (*E*, *A* and the shape of the most appropriate *f*(*α*)-function) of this process by means of thermogravimetric data, using different calculation procedures.

## Methods

### Materials

The materials used for thermal degradation chitosan obtained from crab shells were commercially obtained from SIGMA-ALDRICH (Cat. No. C3646), deacetylated with ≥75% and were used without further purification. Before using, chitosan was vigorously grounded in agate mortar and dried in air at 60°C for 4 hours.

### Thermal analysis

The thermogravimetrical measurements (TG-DTG-DTA) were carried out in a flow of synthetic air at a rate of 25 cm^3^ min^–1^ under non-isothermal conditions on an instrument STA 449 F3 Jupiter (Nietzsch, Germany) with its high temperature furnace. Samples of about 7 ±0.1 mg mass were used for the experiments varied out at hearing rates of 3, 6, 9, 12, 15 and 18°C min^-1^ up to 800°C. The samples were loaded without pressing into an open 6 mm diameter and 3 mm high platinum crucible, without using of a standard reference material. The TG, DTG and DSC curves were recorded simultaneously with 0.1 mg sensitivity.

### Theoretical approach and calculation procedures

TG/DTG technique is very useful for the determination of decomposition temperature, decomposition steps and kinetics triplet for solid substances. The kinetics of thermal degradation reactions is described with various equations taking into account the special features of their mechanisms. The reaction rate can be expressed through the degree of conversion *α*, according to the Equation (1):

(1) $$\alpha =\frac{m_{\text{i}}-m_{\text{t}}}{m_{\text{i}}-m_{\text{f}}}\text{,}$$

where: *m*_i_*m*_f_ and *m*_t_ are the initial, final and current sample mass at the moment *t*, respectively. The rate of many condensed phase reactions can be conveniently parameterized as a function of the temperature *T*, and the extent of the reaction conversion *α*. In non-isothermal kinetics of heterogeneous condensed phase reactions, it is usually accepted that the reaction rate is given by [23-25]:

(2) $$\frac{\text{d}\alpha }{\text{d}t}=q\frac{\text{d}\alpha }{\text{d}T}=A\exp\left(-\frac{E}{RT}\right)f(\alpha )\text{,}$$

where: *α* is the degree of conversion, *T* – absolute temperature, *t* – time, *f*(*α*) – the differential conversion function, *R* – the gas constant (8.314 Jmol^-1^ K^-1^), *q* is the linear constant heating rate *q* = d*T*/d*t* and *A* and *E* are the pre-exponential factor or frequency factor and the activation energy respectively, given by the Arrhenius equation. The conversion function f(*α*) for a solid-state reactions depend on the reaction mechanism and can generally be considered to be as follows [32]:

(3) $$f(\alpha )=\alpha^{\text{m}}{\left(1\;–\alpha \right)}^{\text{n}}{\left[-\text{ln}(1\;–\alpha )\right]}^{\text{p}}\text{,}$$

where *mn* and *p* are empirically obtained exponent factors, one of them always being zero. The combination of different values of *mn* and *p* make it possible to describe various probable mechanisms [23,25,32].

After substitution of Eq. (3) in Eq. (2), separation of variables and integration, the following general equation was obtained:

(4) $$\int_{0}^{\alpha }\frac{\text{d}\alpha }{\alpha^{\text{m}}{\left(1-\alpha \right)}^{\text{n}}{\left[-\ln\left(1-\alpha \right)\right]}^{\text{p}}}=\frac{A}{q}\int_{0}^{\text{T}}\text{exp}\left(-\frac{E}{RT}\right)\text{d}T\text{,}$$

The solution of left-hand side integral depend on the explicit expression of the function *f*(*α*) and are denoted as *g*(*α*). The formal expression of the function *g*(*α*) depend on the conversion mechanism and its algebraic expression. The latter usually represents the limiting stage of the reaction – the chemical reactions; random nucleation and nuclei growth; phase boundary reaction or diffusion. The algebraic expression of functions of the most common reaction mechanisms operating in solid-state reactions are presented in some papers [23-25,27,29].

The right-hand side integral or so called “temperature integral” in Eq. (4) has no exact analytical solution, and can be numerically calculated, making the substitution *u* = *E*/(*RT*) and using the relation:

(5) $$\int_{\text{u}}^{\infty }{\text{e}}^{-\text{u}}u^{-\text{b}}\text{d}u≅u^{1-\text{b}}{\text{e}}^{\text{u}}\sum_{\text{n}=0}^{\infty }\frac{{\left(-1\right)}^{\text{n}}b^{\text{n}}}{{\text{u}}^{\text{n}+1}}\text{,}$$

this difficulty can be overcome [28,33,34] may be rewritten as follows:

(6) $$g\left(\alpha \right)=\frac{AE}{qR}\int_{\text{u}}^{\infty }\frac{{\text{e}}^{-\text{u}}}{{\text{u}}^{2}}\text{d}u=\frac{AE}{qR}p\left(u\right)\text{,}$$

where *p*(*u*) is the exponential integral. Several author [35-38] suggested different ways to solve this exponential integral.

### Coats-Redfern calculation procedure

Coats and Redfern [35] making some approximations, proposed the next linear equation:

(7) $$\ln\frac{g\left(\alpha \right)}{T^{2}}=\ln\frac{AR}{qE}-\frac{E}{RT}+\ln\left(1-\frac{2RT}{E}\right)≅\ln\frac{AR}{qE}-\frac{E}{RT}$$

For most values of *E* and for the temperature range over which reactions generally occurs, and because 2*RT*/*E* is much lower than unity, we have reason to write the right side of Eq. (7). If the correct *g*(*α*) function is used, a plot of ln*g*(*α*)/*T*^2^ against 1/*T* should give a straight line from which the values of the activation energy *E* and the pre-exponential factor *A* in Arrhenius equation can be calculated. They can be calculated from the slope and intercept respectively. The model that gives the best linear fit is selected as the chosen model. The integral method of Coats-Redfern has been mostly and successfully used for studying of the kinetics of dehydration and decomposition of different solid substances when 20 < *E*/*RT* < 60 [23,24,26-28]. This approach is applied and for single TG curves.

### Iso-conversional method

Iso-conversional methodology in non-isothermal experiments is recommended from ICTAC kinetics committee [39]. Iso-conversional methodology in non-isothermal experiments assumes that for a given degree of conversion α, the reaction mechanism does not depend on the heating rate. In this respect Kissinger-Akahira-Sunose [28,40] proposed own model-free method and the next linear equation:

(8) $$\ln\left(\frac{q}{T^{2}}\right)=\ln\left[\frac{AR}{g\left(\alpha \right)E}\right]-\frac{E}{RT}\text{,}$$

For each conversion degree *α*, the linear plot of ln(*q*/*T*^2^) versus 1/*T* enables *E* and ln*AR*/*g*(*α*)*E*to be determined from the slope and the intercept respectively. If the reaction model *g*(*α*) is a priory known, the corresponding pre-exponential factor can be calculated for each conversion degree. If the reaction model *g*(*α*) is unknown, the following equation may be used to estimate the most probably mechanism function [23,25]:

(9) $$\ln g\left(\alpha \right)=\left[\ln\frac{AE}{R}+\ln\frac{{\text{e}}^{-u}}{u^{2}}+\ln h\left(u\right)\right]-\ln q\text{,}$$

where *h*(*u*) is expressed by the fourth Senum and Yang [41] approximation formulae:

(10) $$h\left(u\right)=\frac{u^{4}+18u^{3}+86u^{2}+96u}{u^{4}+20u^{3}+120u^{2}+240u+120}$$

Plotting ln*g*(*α*) versus ln*q* and using linear regressive of least square method, if the mechanism studied conforms to certain *g*(*α*) function, the slope of the straight line should be equal to −1.0000 and the linear correlation coefficient *R*^2^ should be equal to unity. Obviously, the values of *E* and *A* do not influenced on the shape of this obtained straight lines.

The Ozawa-Flynn-Wall and Popescu integral methods [42-44], based on Doyle’s approach [28] was not used because it gives similar results to the KAS calculation procedure.

From the theory of activated complex (transition state) of Eyring [23,24,26,27] the following general equation may be written:

(11) $$A=\frac{e\chi k_{\text{B}}T_{\text{p}}}{h}\exp\left(\frac{\Delta S^{\neq }}{R}\right)\text{,}$$

where *e* = 2.7183 is the Neper number, *χ* is the transition factor, which is unity for monomolecular reactions, *k*_B_ is the Boltzmann constant, *h* is the Plank constant and *T*_p_ is the peak temperature of DTG curve. The change of the entropy at the formation of the activated complex from the reagent may be calculated according to the formula [23,24,26,27]:

(12) $$\Delta S^{\neq }=R\ln\frac{Ah}{e\chi k_{\text{B}}T_{\text{p}}}$$

Since

(13) $$\Delta H^{\neq }=E-RT_{\text{p}}\text{,}$$

the changes of the enthalpy Δ*H*^≠^ and Gibbs free energy Δ*G*^≠^ for the activated complex formation can be calculated, using well known thermodynamic equation:

(14) $$\Delta G^{\neq }=\Delta H^{\neq }-T_{\text{p}}\Delta S^{\neq }$$

The values of Δ*S*^≠^, Δ*H*^≠^ and Δ*G*^≠^ were calculated at *T* = *T*_p_ (*T*_p_ is the peak temperature at the corresponding stage), since this temperature characterizes the highest rate of the process, and therefore, is its important parameter [23,24,26,27].

### Estimation of lifetime

Lifetime estimations are very useful in the development or selection of polymers for different applications. The lifetime is usually determined by accelerated aging, like air oven aging studies, which require long time periods. Chitosan, being natural biopolymer have own lifetime, which value is very important parameter. The apparent kinetic parameters calculated according above described manner has been used to calculate the value of lifetime for chitosan. The estimated lifetime or the time accelerating ageing *t*_f_ of a chitosan to failure has been defined as the time when the mass loss reaches 5 mass%, i.e. *α* = 0.05 [28,45-47] and can be estimated by the following equation:

(15) $$t_{\text{f}}=\frac{0.0513}{A}\exp\left(\frac{E}{RT}\right)\;\text{if}\;n=1$$

or

(16) $$t_{\text{f}}=\frac{\left(1-0.95^{1-\text{n}}\right)}{A\left(1–n\right)}\exp\left(\frac{E}{RT}\right)\text{,}\;\text{at}\;n\neq 1$$

where the value of the reaction order *n* is obtained previously. With these equations, the time to equivalent damage at different temperatures can be calculated.

## Results and discussion

**Figure 2:**
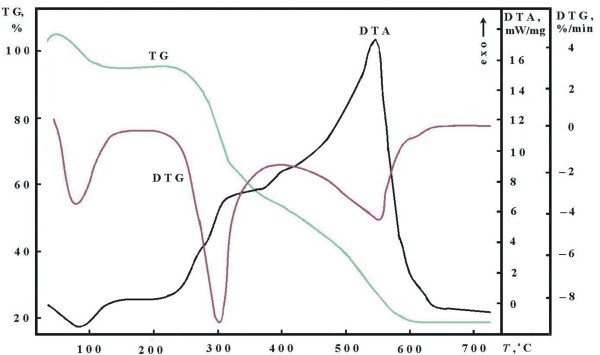
**TG, DTG and DTA curves, obtained for thermal degradation of chitosan at heating rate of 15°C min**^**-1**^**.**

The TG, DTG and DTA curves, obtained by the thermal degradation of chitosan at heating rate of 15°C min^-1^ are presented on Figure 2.

Two steps can be observed in the TG-curve: the first one at about 90°C connected with 5.8% mass loss was accompanied by endothermic effect and was attributed to the evaporation of water absorbed in the inner polymer. The second one, beginning at about 245°C and ending at 580°C was connected with 78.5% mass loss and was indicated for vaporization and burning of volatile compounds produced from the thermal degradation of polymeric chain. The pyrolysis of polysaccharides structure starts by a random split of the glycosidic bonds followed by a further decomposition to form acetic and butyric acids and a series of lower fatty acids where C2, C3 and C6 predominate [14]. At temperatures above 380°C, significant change was observed in the course of the TG-curve. It is may be due to the change of the structure of the material and the change of the mechanism of its thermal degradation process. In the DTG and DTA-curves, two peaks were observed – at 294.5 and 544.5°C, respectively. These two stages are strongly exothermic. The first stage ending at 400°C is connected with 43.5% mass loss while the second one – with 35% mass loss. According to some authors [12-14] the first stage is connected with deacetylation and depolymeryzation of chitosan. The second one corresponds to the residual cross-liked degradation chitosan [14].

**Figure 3:**
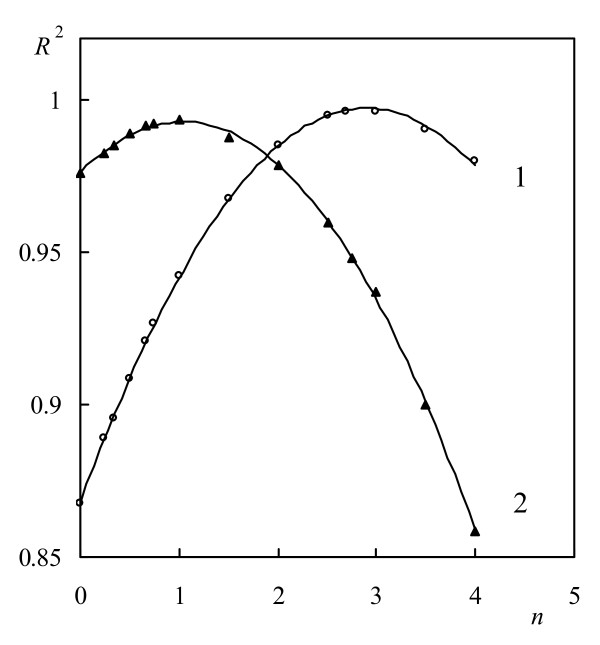
**Dependences of the coefficient of linear regression *R* **^**2**^**on the values of *n* in *F* **_**n**_**type functions for both stages of thermal degradation.**

Using Coats-Redfern calculation procedure and on the basis of different f(*α*) functions, which algebraic expression is presented in our previous work [48] were plotted the dependences of ln(g(*α*)/*T*^2^) on 1/*T* according to Eq. (7). The values of the coefficient of linear regression *R*^2^ strongly depends form the shape of used f(*α*) function. On Figure 3 are presented the dependences of *R*^2^ versus *n* for *F*_n_ mechanism functions with different values of *n* at a heating rate of 15°C min^-1^.

As can be seen from Figure 3, the both stages of thermal degradation strongly depends from the values of *n* and are characterized with different most appropriate mechanism functions. The curves presented can be successfully described by empirical polynomials of second power and different coefficients. Differentiating and nullifying these polynomials, the value of *n* at which *R*^2^ has maximum value can be calculated. For the first stage of thermal decomposition was established that the maximal value of *R*^2^ was obtained at *n* = 3, and for the second stage – at *n* = 1 respectively. It means that, the kinetics of the both stages is different and the shapes of the most appropriate mechanism function are different. The same tendency was established for the all used heating rates. The values of *E* and *A* for the both stages of the thermal degradation of chitosan obtained at different heating rates are presented in Table 1 for comparison.

**Table 1:** Effect of the heating rate on the kinetic parameters for the thermal decomposition of chitosan calculated according Coats-Redfern procedure

**Parameters**	**Heating rate (K min**^**–1**^**)**						**Average**
	**3**	**6**	**9**	**12**	**15**	**18**	
**First stage**							
*E* (kJ mol^-1^)	137.8	136.4	131.3	126.4	132.8	112.8	**129.6**
*A* (min^-1^)	8.10×10^12^	7.33×10^12^	2.23×10^12^	8.25×10^11^	2.41×10^12^	2.90×10^10^	**3.49×10**^**12**^
**Second stage**							
*E* (kJ mol^-1^)	100.2	99.8	102.1	101.5	105.7	102.4	**102.0**
*A* (min^-1^)	7.38×10^5^	9.13×10^5^	1.32×10^6^	1.24×10^6^	2.12×10^6^	1.08×10^6^	**1.23×10**^**6**^

As can be seen from Table 1 the average value of *E* and *A* for the first stage of the thermal degradation of chitosan is higher than these for the second one. Because the obtained value of the apparent activation energy *E* for the second stage are near to 100 kJ mol^-1^, may be say that the kinetic of this stage is diffusion controlled process.

**Figure 4:**
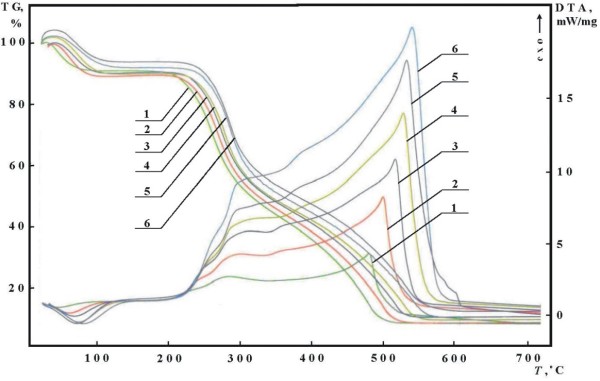
**TG and DTA-curves, obtained at different heating rates: 1 – 3; 2 – 6; 3 – 9; 4 – 12; 5 – 15 and 6 – 18°C min**^**-1**^**.**

The TG and DTA-curves obtained at different heating rates are presented in Figure 4 for comparison.

As can be seen from Figure 4, the TG and DTA-curves are shifted to higher temperatures with the increase of the heating rates. Furthermore, two exo-effects were observed, the second of them stronger. These effects correspond to a step which abruptly changes its course at temperatures above 400°C. It means that the kinetic model describing the thermal decomposition (the algebraic expression of *f*(*α*)-function) will also change.

**Figure 5:**
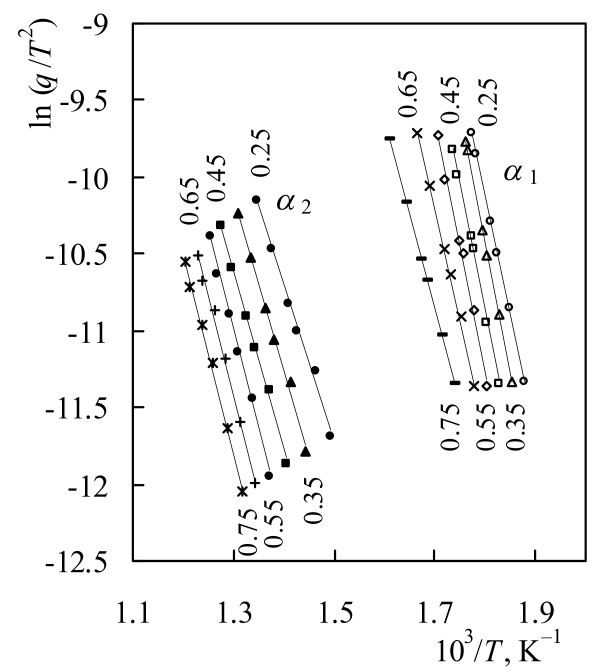
**Iso-conversional plots at various conversion degree of chitosan drawn according KAS calculation procedure for: first stage – *α* **_**1**_**and second stage – *α* **_**2**_**respectively.**

To perform the kinetic computations on the thermal analytical data, the Kissinger-Akahira-Sunose calculation procedure was used, recommended from ICTAC kinetics committee [39]. Taking subsets of the TG-curves at certain values of *α* and taking the corresponding temperatures, the dependence of ln(*q*/*T*^2^) on 1/*T* was drawn according to Eq.(8) for both stages (Figure 5).

The apparent activation energy *E* was directly evaluated from the slope of these plots and frequency factor *A* – from the cut-off from the ordinate axis respectively.

**Figure 6:**
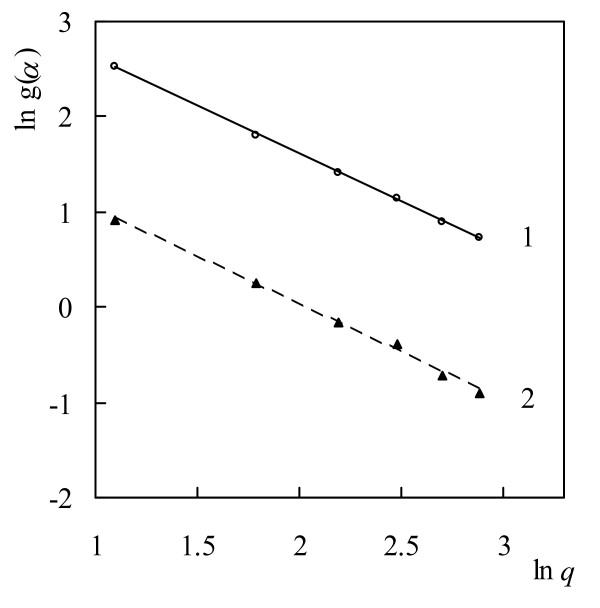
**Plots of ln[ *g* ( *α* )] against ln *q* for thermal degradation of chitosan: 1 – first stage and 2 – second stage.**

For determination of the most probable mechanism function *g*(*α*), Eq.(9) was used. Plotting ln[*g*(*α*)] against ln*q* with different algebraic expressions of *g*(*α*)-function and using a linear regression of the least squares method, we were looking for a straight line with slope equal to −1.0000 for which the linear correlation coefficient *R*^2^ is close to unity. The graphical dependence of ln[*g*(*α*)] against ln*q* for both stages is presented in Figure 6.

Lines with slope equal to −1.0000 and linear correlation coefficient *R*^2^ close to unity were obtained with F_n_ mechanism functions at different values of *n*. For the first stage, it was established that *n* is equal to 3.0 and for the second stage - to 1.0 respectively. A reaction order *n* > 2 is mathematically equivalent to Gamma distribution of frequency factor. The interpretation of the high values of reaction order has been discussed in the literature [49]. However, the reason why this parameter has such a high value in relation to the described phenomena has not been completely explained.

The values of the apparent activation energy *E* and pre-exponential factor *A* calculated for both stages of the thermal degradation of chitosan are presented in Table 2. The values of the apparent activation energy are in good agreement with earlier reported ones [13,14,49]. Using Eqs. (12), (13) and (14), the values of the change of entropy Δ*S*^≠^, enthalpy Δ*H*^≠^ and the Gibbs free energy Δ*G*^≠^ for the formation of the activated complex from the reagent were calculated. The change of the activation enthalpy *H*^≠^ shows the energy difference between the reagent and activated complex. If this difference is small, the formation of the activated complex is favored because the potential energy barrier is low. The change of the Gibbs free energy Δ*G*^≠^ reveals the total energy increase of the system at the approach of the reagents and the formation of the activated complex.

**Table 2:** Kinetic parameters of the thermal degradation of chitosan

** *α* **	** *E* (kJ mol**^**–1**^**)**	** *A* (min**^**-1**^**)**	**–Δ *S* **^**≠**^**(J mol**^**–1**^** K**^**–1**^**)**	**Δ *H* **^**≠**^**, (kJ mol**^**–1**^**)**	**Δ *G* **^**≠**^**, (kJ mol**^**–1**^**)**
**First stage**					
0.25	126.0	3.34×10^11^	70.8	121.9	156.6
0.35	137.6	5.32×10^12^	47.8	133.5	156.9
0.45	136.2	3.92×10^12^	50.3	132.1	156.8
0.55	133.6	2.30×10^12^	54.7	129.5	156.3
0.65	121.5	1.60×10^11^	76.9	117.4	155.1
0.75	101.0	1.78×10^9^	114.3	96.9	153.0
**Average**	**126.0**	**2.01×10**^**12**^	**69.1**	**121.9**	**155.8**
**Second stage**					
0.25	83.3	3.73×10^4^	207.6	76.9	235.9
0.35	92.3	2.36×10^5^	192.3	86.0	233.2
0.45	97.9	6.18×10^5^	184.2	91.6	232.7
0.55	105.8	2.27×10^6^	173.4	99.5	232.3
0.65	107.3	2.70×10^6^	172.0	100.9	232.6
0.75	108.0	2.99×10^6^	171.2	101.6	232.7
**Average**	**99.1**	**1.47×10**^**6**^	**183.4**	**92.7**	**233.3**

As can be see from Table 2, the average value of the apparent activation energy *E* and pre-exponential factor *A* in the Arrhenius equation is higher for the first stage of the thermal degradation of chitosan. The values of the pre-exponential factor for a solid phase reactions are expected to be in a wide range (six or seven orders of magnitude), even after the effect of surface area is taken into account [24]. For first order reactions, the pre-exponential factor may vary from 10^5^ to 10^16^ min^-1^. The low factors will often indicate a surface reaction, but if the reactions are not dependent on surface area, the low factor may indicate a “tight” complex. The high factors will usually indicate a “loose” complex [23]. Even higher factors (after correction for surface area) can be obtained for complexes having free translation on the surface. Since the concentrations in solids are not controllable in many cases, it would have been convenient if the magnitude of the preexponential factor indicated for reaction molecularity. However, this appears to be true only for non-surface-controlled reactions having low (<10^8^ min^-1^) pre-exponential factors. Such reactions (if elementary) can only be bimolecular.

The change of entropy for the formation of the activated complex from the reagent Δ*S*^≠^ reflects how close the system is to its own thermodynamic equilibrium. Lower activation entropy means that the material has just passed through some kind of physical or chemical rearrangement of the initial structure, bringing it to a state close to its own thermodynamic equilibrium. In this situation, the material shows little reactivity, increasing the time necessary to form the activated complex. On the other hand, when high activation entropy values are observed, the material is far from its own thermodynamic equilibrium. In this case, the reactivity is higher and the system can react faster to produce the activated complex, and consequently, short reaction times are observed. In particular, for example, the negative values of Δ*S*^≠^ would indicate that the formation of activated complex is connected with decrease of entropy, i.e. the activated complex is “more organized” structure compared to the initial substance and such reactions are classified as “slow” [23]. The negative values of Δ*S*^≠^ obtained for the second stage of the thermal degradation of chitosan showed that its structure is far from its own thermodynamic equilibrium, comparing with the second one.

On Table 3 are given for comparison the kinetic parameters, characterizing thermal degradation of chitin and chitosan.

**Table 3:** **Comparison of the kinetic parameters obtained with the most probable mechanism function g( *α* ) for non-isothermal degradation of chitin and chitosan**

**Parameter**	**Chitin**		**Chitosan**	
	**First stage**	**Second stage**	**First stage**	**Second stage**
*E* (kJ mol^–1^)	154.0	114.8	126.0	99.1
*A* (min^-1^)	2.51×10^14^	5.36×10^7^	2.01×10^12^	1.47×10^6^
–Δ*S*^≠^ (J mol^–1^ K^–1^)	29.7	148.3	69.1	183.4
Δ*H*^≠^ (kJ mol^–1^)	149.7	108.5	121.9	92.7
Δ*G*^≠^ (kJ mol^–1^)	165.0	220.6	155.8	233.3

As can be seen from Table 3 chitin is more stable than chitosan. The values of the apparent activation energy and pre-exponential factor for both stages of thermal degradation of chitin are higher than these of chitosan. The same tendency was established from other authors [12,49]. The negative values of Δ*S*^≠^ for both compounds showed that the formation of the activated complex from the reagents is connected with a decreasing of entropy, i.e. the activated complex is “more organized” structure and the formation process may be classify as “slow” [23].

**Figure 7:**
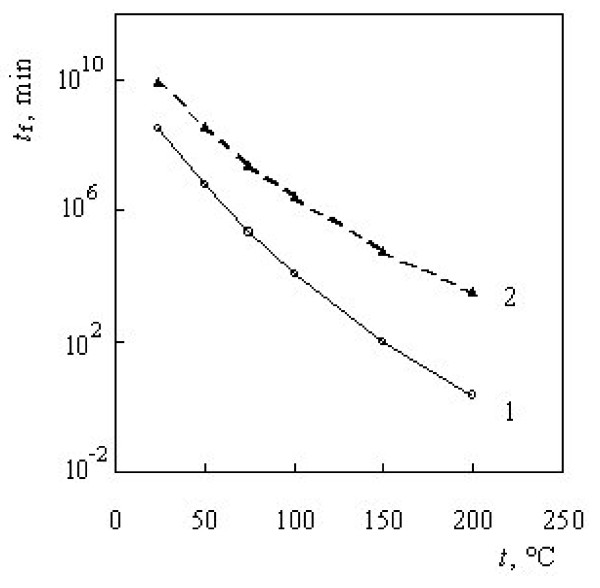
Dependence of the lifetime on the temperature at 5% conversion for: 1 – first stage and 2 – second stage of the thermal degradation of chitosan.

The curves representing the dependence of lifetime on the temperature at 5% conversion for both stages of thermal degradation of chitosan are shown in Figure 7.

It is obvious from Figure 7 that the lifetime is a parameter strongly depending on the temperature and decreases exponentially with the increase of the temperature. The lifetime is more sensitive concerning the temperature for the first stage of the thermal decomposition of chitin. The same tendency was established for the lifetime of chitin [48].

## Conclusion

Chitosan have excellent properties such as hydrophilicity, biocompatibility, biodegradability, antibacterial, non-toxicity, adsorption application. The thermal degradation of chitosan occurs in two stages. The kinetics and mechanism of the thermal decomposition reaction were established using iso-conversional calculation procedure. The most probable mechanism function for both stages is determined and it was best described by kinetic equations of *n*^-th^ order (*F*_n_ mechanism). For the first stage, it was established that *n* is equal to 3.0 and for the second stage – to 1.0 respectively. The values of the apparent activation energy *E*, pre-exponential factor *A* in Arrhenius equation, as well as the changes of entropy Δ*S*^≠^, enthalpy Δ*H*^≠^ and free Gibbs energy Δ*G*^≠^ for the formation of the activated complex from the reagent are calculated.

## **Competing interests**

The authors declare that they have no competing interests.

## **Authors’ contributions**

LV has formulated the research idea and planed the experiment. DZ has carried out the collection of data. VG has processed results and has prepared the figure and tables and has finalized the manuscript. All authors read and approved the final manuscript.
